# The spectrum of functional neurological disorders: A retrospective analysis at a tertiary hospital in South Africa

**DOI:** 10.4102/sajpsychiatry.v27i0.1607

**Published:** 2021-04-19

**Authors:** Lavanya Naidoo, Ahmed I. Bhigjee

**Affiliations:** 1 Department of Neurology, Nelson R Mandela School of Medicine, University of Kwazulu-Natal, Durban, South Africa

**Keywords:** functional neurological disorders, conversion disorder, somatisation disorder, psychiatric diagnosis, African ethnicity

## Abstract

**Background:**

Functional neurological disorders (FNDs) are commonly encountered in practice; however, there is a paucity of data in Africa.

**Aim:**

To identify and describe the clinical profile of patients presenting with FNDs, underlying medical and psychiatric diagnoses and review the investigation and management of these patients.

**Setting:**

Inkosi Albert Luthuli Central Hospital (IALCH), a tertiary-level hospital in Durban, South Africa.

**Methods:**

A retrospective chart review and descriptive analysis were performed over a 14-year period (2003–2017) on cases meeting the study criteria.

**Results:**

Of 158 subjects, the majority were female (72.8%), had a mean age of 32.8 years, were single (63.3%), unemployed (56.3%) and of black African ethnicity (64.6%). The most common clinical presentation was sensory impairment (57%) followed by weakness (53.2%) and seizures (38.6%). Inconsistency was the most frequent examination finding (16.5%). Medical conditions were identified in half of the study population (51.3%), with hypertension (22.2%) and human immunodeficiency virus (HIV) (17.2%) being most common. Of patients with a psychiatric diagnosis (55.1%), 25.3% had depression. Magnetic resonance imaging (MRI) was the most frequently performed investigation (36.1%). The majority of patients received psychotherapy (72%) and most had not shown improvement (55.3%) at a median follow-up of 2 months, whilst 17% had deteriorated.

**Conclusion:**

Functional neurological disorders were most frequently diagnosed in young unmarried females, of black African ethnicity. Family history, personal exposure to a neurological illness and certain socioeconomic factors may be potential risk factors. Sensory impairment was the most common clinical phenotype. Further studies are needed to better understand and manage FNDs in the South African context.

## Introduction

Functional neurological disorders (FNDs) refer to the presence of neurological symptoms in the absence of organic neurological disease and have been documented for centuries under such terms as ‘hysteria’.^1,2^

Charcot used the term ‘functional’ to describe those disorders where no anatomical or histological pathology could be demonstrated but function was impaired.^3^ Freud and Janet later argued that the basis for the disorder was a psychological phenomenon.^4^

Terminology has included purely symptomatic diagnoses or syndromes, ‘non-diagnoses’ such as ‘non-epileptic seizures’ and psychogenic, psychosomatic or functional disorders. Psychiatric terms include conversion disorder, somatisation, dissociative motor disorder, hypochondriasis, factitious disorder, Munchausen syndrome and malingering. All carry different connotations with regard to causation and may affect the patient’s perception of their illness.^5^

‘Functional neurological symptom disorder’ was added to the Diagnostic and Statistical Manual of Mental Disorders, Fifth Edition (DSM-V) criteria in 2013 and distinguished functional disorders from intentional disorders such as malingering or factitious disorder, although this distinction is debated.^6,7^

‘Functional neurological symptom disorder’ is a broad term implying a change in function rather than structure and has become a preferred term, as it avoids the insinuation that ‘everything is in the patient’s mind’ and uncertainty as to whether the problem is neurological or psychological.^8^

Functional neurological disorders are commonly encountered in neurology clinics and may cause levels of disability and impaired quality of life similar to multiple sclerosis and Parkinson’s disease.^9,10^

One-third of neurological outpatients experience symptoms regarded as ‘not at all’ or ‘only somewhat’ explained by disease,^11^ and approximately 50% have functional symptoms even if it is not the main complaint.^8^

Neurologists and non-neurologists alike are often faced with the challenge of inconsistent examination findings, uncertainty as to whether there is co-existent organic pathology and the need to investigate these patients, sometimes at unnecessary cost. In resource-limited settings with high burdens of disease such as South Africa, the need to practise evidence-based, cost-effective medicine is essential.

Although a number of international studies have described the spectrum of FNDs, there is a paucity of data from South Africa and recent literature from Africa. Studies that describe the spectrum of FNDs were either conducted over 50 years ago or if more recent have focussed primarily on psychogenic non-epileptic seizures (PNES) and included relatively small sample sizes.^12,13^

The aim of this study was to document the experience at a tertiary-level referral centre in Durban, South Africa, and identify the clinical profile, investigation and management of patients with FND presenting to the Inkosi Albert Luthuli Central Hospital (IALCH) over a 14-year period. Inkosi Albert Luthuli Central Hospital serves a population of approximately 18 million people. Patients attending public health facilities in Kwazulu-Natal (KZN) (approximately 79.5% of the population) are mostly of black African ethnicity (90.3%) reflecting local demographics.^14,15^

Addressing the relative lack of local data on this subject may lay the foundation for future research in this important yet neglected field in Southern Africa.

## Methods

A retrospective chart review was conducted on patients presenting to IALCH with a diagnosis of FND between 2003 and 2017. The tenth revision of the International Statistical Classification of Diseases and Related Health Problems (ICD) coding system was used to identify cases. ICD codes F44 – dissociative (conversion disorders), F45 – somatoform disorders, F51 – non-organic sleep disorders and F99 – mental and behavioural disorders were included. Data were extracted by using the Clickview software program, a data extraction program that is able to select patient charts via specific patient numbers according to ICD-10 codes entered. Charts were accessed via the Meditech software system. Patients with an organic neurological disease that may have explained their symptoms and those with insufficient clinical information were excluded. A total of 158 patients were analysed.

A data collection tool created by the authors was used to document demographic data, clinical presentation, examination findings, organic disease, psychiatric illness if known or diagnosed during admission, investigations performed, management and follow-up.

## Statistical analysis

Descriptive analysis was used to address all the objectives. Continuous variables were summarised by using mean, standard deviation and range, whilst categorical variables were tabulated as frequency and percentage. Categorical variables were analysed by using the Chi-square test, and non-parametric testing was performed for continuous variables. IBM SPSS version 25 was used to analyse the data.

### Ethical considerations

The Ethics committee of the Faculty of Health Sciences at the University of Kwazulu-Natal, Durban, South Africa, granted the ethical approval for the study, reference number: BE042/18.

## Results

During the 14-year study period, 158 subjects with FND were analysed. Patient demographics and characteristics are listed in Table 1.

**TABLE 1:** Patient demographics and clinical characteristics.

Variable	Frequency, total 158			
	Mean	s.d.	*n*	%
**Age**	**32.8**	**13.2**	**-**	**-**
**Gender**				
Female	-	-	115	72.8
Male	-	-	43	27.2
**Race**				
Black people	-	-	102	64.6
Indian	-	-	41	25.9
White people	-	-	12	7.6
Not captured	-	-	3	1.9
**Employment status**				
Unemployed	-	-	89	56.3
Employed	-	-	58	36.7
Not captured	-	-	11	7.0
**Relationship status**				
Single	-	-	100	63.3
Married	-	-	46	29.1
Partner	-	-	4	2.5
Not captured	-	-	8	5.1
**Religion**				
Christian	-	-	9	5.7
Muslim	-	-	4	2.5
Hindu	-	-	3	1.9
Not captured	-	-	141	89.2
**Organic disease**				
Hypertension	-	-	18	22.2
HIV	-	-	14	17.2
Asthma	-	-	13	16.0
Diabetes mellitus	-	-	7	8.6
Not captured†	-	-	29	35.8

s.d., standard deviation.

† , Only the four most common organic conditions were analysed.

### Neurological symptoms and signs

Sensory disturbance, *n* = 90/158 (57%), was the most common clinical complaint followed by weakness, *n* = 84/158 (53.2%), and seizures, *n* = 61/158 (38.6%; Table 2).

**TABLE 2:** Neurological presentations of functional neurological disorders.

Phenotype	Frequency, total 158	
	*n*	%
**Sensory disturbance**	**90**	**57.00**
Hemisensory loss	32	35.60
Level	12	13.30
Dermatomal	1	1.11
Other†	8	8.90
Not captured	37	41.00
**Weakness**	**84**	**53.20**
Paraparesis	31	36.90
Hemiparesis	28	33.30
Monoparesis	11	13.10
Quadriparesis	9	10.70
Triparesis	1	1.20
Not captured	4	4.80
**Seizures**	**61**	**38.60**
Generalised	33	54.00
Focal	22	36.00
Not captured	6	9.8 0
**Abnormal gait**	**52**	**32.90**
**Movement disorder**	**22**	**13.90**
Tremor	18	82.00
Other‡	4	18.00

† , Paraesthesias, facial numbness and hyperalgesia.

‡ , Athetoid movements of upper and lower limbs (*n* = 1), bizarre movements of all four limbs (*n* = 1), dystonia (*n* = 1) and hemiballismus (*n* = 1).

Hemisensory loss was found in a third of subjects, *n* = 32/90 (35.6%), that reported sensory disturbances. When noted, the sensation was reported to split the midline including vibration sense in six patients. Other sensory complaints were paraesthesias (*n* = 6), facial numbness (*n* = 1) and hyperalgesia (*n* = 1).

Eighty-four (53.2%) patients of the total study population complained of weakness. Paraparesis accounted for *n* = 31/84 (36.9%) of presentations with weakness, followed by hemiparesis, *n* = 28/84 (33%) (Table 2). In patients who presented with hemiparesis, most were left-sided, *n* = 15/28 (53.6%), and when handedness was documented in these patients, all were noted to be right-handed. Thirty-three per cent (*n* = 3) of the nine patients who presented with quadriparesis reported associated incontinence. This was higher than the percentage of patients who had incontinence in association with other forms of weakness (hemiparesis: *n* = 4/27, 14.8%; paraparesis: *n* = 4/31, 13%; monoparesis: *n* = 1/11, 9.1%; and triparesis: *n* = 0, 0%; Table 6 in supplementary material).

There were 61 patients in whom a diagnosis of psychogenic non-epileptic seizures was documented. Generalised seizures, *n* = 33/61 (54%), were more common than focal seizures, *n* = 22/61 (36%; Table 2). Other documented findings during seizures were being awake (*n* = 6), long duration (*n* = 5), no postictal phase (*n* = 1), hyperventilation (*n* = 1) and high numbers seizures (*n* = 2).

A family history of seizures was present in 19.7% (*n* = 12/61) of patients who presented with seizures themselves, a significantly higher proportion than patients without seizures and a positive family history, *n* = 2/97 (19.7% vs. 2.1%; *p* < 0.001). One patient with seizures was noted to live with a known epileptic patient, although not related. Seven patients were admitted for long-term electroencephalographic (EEG) monitoring of seizures, and all studies were normal apart from one documenting a single epileptic seizure amongst many other non-epileptic seizures. Anti-epileptic drugs were stopped in three patients and continued in three patients. Data on treatment discontinuation were not captured for the rest of the sample.

Other presenting complaints or in addition to the above are listed in Table 3.

**TABLE 3:** Other presenting complaints.

Complaint	Frequency, total 158	
	*n*	%
Headache	19	12.00
Visual loss	11	7.00
Gastrointestinal symptoms	8	5.10
Neck pain	8	5.10
Body/limb pain	8	5.10
Lower back pain	7	4.40
Dizziness	7	4.40
Diplopia	6	3.80
Mutism	5	3.16
Memory loss	5	3.16
Vertigo	3	1.90
Jaw pain	3	1.90
Facial pain	3	1.90
Dysphasia	2	1.27
Cramps	2	1.27
Hypersomnolence	2	1.27
Insomnia	2	1.27
Dysarthria	1	0.63
Syncope	1	0.63
Falls	1	0.63
Imbalance	1	0.63
Eye pain	1	0.63
Chest pain	1	0.63
Tight chest	1	0.63
Menorrhagia	1	0.63
Not captured	49	31.00

Inconsistency was the commonest clinical finding supportive of FND, *n* = 26/158 (16.5%; Table 4). Patients were also reported to have ‘poor effort’ (*n* = 8), hesitancy (*n* = 2), appear anxious (*n* = 2), uncooperative (*n* = 2), resistant (*n* = 2) and tearful (*n* = 1) during examinations.

**TABLE 4:** Clinical signs suggestive of functional neurological disorders.

Sign	Frequency, total 158	
	*n*	%
Inconsistency	26	16.5
Intermittency	25	15.8
Variability	21	13.3
Hoover’s sign	9	5.7
Distractible	9	5.7
No ‘positive functional sign’ documented	68	43.0

### Psychiatric diagnoses

A psychiatric diagnosis was present in 55% of patients (*n* = 87/158). Depression was the commonest psychiatric diagnosis documented overall (Table 5). Twenty one percent (*n* = 18/87) were diagnosed before admission. Of those diagnosed pre-admission, *n* = 7/18 (38.9%) had depression, *n* = 6/18 (33.3%) had both anxiety and depression and *n* = 4/18 (22%) had other diagnoses. When psychiatric diagnoses were made after admission (*n* = 66), 15 (22.7%) were diagnosed with depression, 1 with anxiety and 14 (21%) with other diagnoses.

**TABLE 5:** Psychiatric diagnoses in patients with functional neurological disorders.

Diagnosis	Frequency; total 87	
	*n*	%
Depression	22	25.3
Depression and anxiety	5	5.7
Anxiety	1	1.1
Other psychiatric diagnoses†	19	22.0
Not captured	40	46.0

† , Somatisation disorder, post-traumatic stress disorder, psychosis, dysthymic disorder, panic attacks, histrionic personality disorder and narcissistic personality disorder.

**TABLE 6:** Incontinence in association with forms of weakness.

Form of weakness	Total (*n*)	Frequency of incontinence	
		*n*	%
Quadriparesis	9	3	33.3
Hemiparesis	27	4	14.8
Paraparesis	31	4	13.0
Monoparesis	11	1	9.1
Triparesis	0	0	0.0

Data were insufficient to comment on how diagnoses were made before admission; however, when admitted, either the in-hospital psychologist or the attending neurologist made the diagnosis. Psychiatric symptoms reported included suicidal ideation, hallucinations, excessive agitation and feelings of dissociation such as ‘floating’ or ‘not being in control of a limb shaking’.

### Family history

Family history, when documented, included several co-morbidities in family members: epilepsy (*n* = 18), asthma (*n* = 5), stroke (*n* = 4), hypertension (*n* = 2), multiple sclerosis (*n* = 1), spinal pathology not specified (*n* = 1), brain tumour (*n* = 1), depression (*n* = 1), visual loss (*n* = 1) and hearing loss (*n* = 1) (Table 7). Twelve patients who had relatives with epilepsy presented with seizures. All patients who had relatives with stroke presented with hemiparesis, and the patient who had a relative with spinal pathology presented with paraparesis. Patients with relatives who were asthmatic were all polysymptomatic. Details of these patients are listed in the supplementary material.

**TABLE 7:** Positive family history.

Condition known in family member	Frequency, total 35	
	*n*	%
Epilepsy	18	51.0
Asthma	5	14.3
Stroke	4	11.4
Hypertension	2	5.7
Multiple sclerosis	1	2.9
Brain tumour	1	2.9
Spinal pathology not specified	1	2.9
Depression	1	2.9
Visual loss	1	2.9
Hearing loss	1	2.9

Note: Patients with relatives who were asthmatic were all polysymptomatic:

Patient 1 presented with paraparesis and transient visual loss; patient 2 with seizures, sensory deficits, tightness of chest and abdominal pain; patient 3 with hemiparesis, headache, backache, dysphagia, dysphasia and imbalance; and patient 4 with paraparesis and syncope.

### Psychosocial factors

Stressors documented included domestic (*n* = 4), work-related problems (*n* = 1) and sexual orientation (*n* = 1). Domestic abuse or rape was reported as events preceding the onset of the FND in five patients. Bereavement (*n* = 2), alcohol abuse (*n* = 1), incarceration (*n* = 1) and school failure (*n* = 1) were noted in others. Trauma related to a motor vehicle accident (*n* = 3), surgery (*n* = 1) and a snake bite (*n* = 1) were also identified as preceding events (Table 8).

**TABLE 8:** Psychosocial factors.

Psychosocial factor/stressor	Frequency, total 17	
	*n*	%
Domestic abuse ± stressors	4	24.0
Motor vehicle accident	3	17.6
Bereavement	2	11.8
Work-related stressors	1	5.9
Sexual orientation	1	5.9
Rape	1	5.9
Alcohol abuse	1	5.9
Incarceration	1	5.9
School failure	1	5.9
Surgery	1	5.9
Snakebite	1	5.9

### Investigations

Investigations performed included magnetic resonance imaging (MRI), *n* = 86/238 (36%), computed tomography scan of the brain (CTB), *n* = 68/238 (29%), EEG, *n* = 57/238 (24%), and lumbar punctures (LPs), *n* = 27/238 (11.3%) (Table 9). Abnormalities were documented in 15 MRIs, 13 CTBs, 3 EEGs and 2 LPs. When reported as abnormal, this was uniformly because of incidental findings that did not explain patient symptomatology.

Electroencephalographic findings were abnormal in three patients, of which two did not correlate with the patient’s clinical seizure semiology. The other showed an epileptic seizure captured in association with multiple non-epileptic seizures.

**TABLE 9:** Investigations performed.

Investigation	Frequency, total 238†	
	*n*	%
MRI	86	36.1
CTB	68	29.0
EEG	57	24.0
LP	27	11.3

MRI, agnetic resonance imaging; CTB, computed tomography scan of the brain; EEG, electroencephalography; LP, lumber puncture.

† , Subjects may have had more than one investigation; therefore, total number investigations performed = 238.

Cerebrospinal fluid (CSF) results were abnormal in *n* = 2/27 (7.4%). One was explained by the patient’s HIV status, and the other had an isolated raised protein of 0.61g/dL with 150 red blood cells noted.

### Management

The majority of patients, *n* = 132/184 (72%), either received or were referred for psychotherapy. The use of antidepressants, *n* = 32/184 (17.4%), and physiotherapy, *n* = 20/184 (10.9%), was less common (Table 10).

**TABLE 10:** Management.

Intervention	Frequency, total 184†	
	*n*	%
Psychotherapy	132	72.0
Antidepressants	32	17.4
Physiotherapy	20	10.9

† , Patients may have received more than one intervention; therefore, the total is 184.

### Follow-up

Less than one-third of patients, *n* = 47/158 (30%), attended a follow-up appointment. Symptoms had improved in *n* = 16/47 (34%) of subjects at a median follow-up time of 2 months and had worsened in *n* = 8/47 (17%), but remained static in the majority of patients (Table 11).

**TABLE 11:** Follow-up.

Outcome	Frequency, total 47†	
	*n*	%
Static at follow-up	26	55.3
Better at follow-up	16	34.0
Worse at follow-up	8	17.0

† , Only 47 of 158 patients were followed up.

## Discussion

The present study described the spectrum of FND seen at a tertiary-level hospital in Durban, South Africa. This study documented the commonest clinical presentation of FND in a South African setting, demographics of patients presenting with these disorders, and provided insight into factors that may play a role in the development of the disease. It also demonstrated the lack of adequate management and follow-up of patients with FND and the need for improving patient outcomes (Table 11). The investigations performed on patients with FND were also highlighted. The available laboratory and imaging facilities allowed the authors to study the patients in detail, a situation that is often lacking in resource-poor sub-Saharan countries. These countries are further hamstrung by the shortage of neurologists who play a key role in the diagnosis of FND. Additional challenges are stigma and lack of understanding of FND in the community.

This retrospective study of the spectrum of FND is, to the best of the authors’ knowledge, the largest systematic analysis of FND in Africa to date. Dekker et al.^12^ published a case series in Tanzania with a sample number of 44, whilst earlier studies on the pattern of neurological illness in Nigeria documented FND in 23 of 9359 patients.^13^

In keeping with international studies and other African studies, a female predominance was noted.^12,16,17,18^ The mean age of presentation in this study was younger than in international studies (32.8 years vs. 47.9 years [US] and 43 years [UK])^16,17^ but older than in the 2018 Tanzanian study in which almost 50% of patients with FND were below the age of 20.^12^

Jacob et al.^16^ reported that most patients with FND were married and well-educated. In that study no association was found between FND and employment status. Our investigation found a predominance of unmarried individuals who were unemployed and of black African ethnicity. Two South African studies reported that most patients (77% and 60%) who presented with PNES were of the white ethnic background; however, these studies analysed patients presenting with PNES only, with one of them limited to patients in a private healthcare setting, which is not accessible to the majority of the local population.^18,19^

As suggested in our study, social circumstances contribute to the development of FNDs and their role is thought to be causative in many cases.^5^ Multiple social stressors including physical and sexual abuse, alcoholism, scholastic failure and bereavement were reported amongst subjects over the study period, which is similar to international data.^16^

A South African study of pseudoseizures in patients with neurocysticercosis found 17/29 (58%) female patients had experienced sexual or physical abuse and problems related to alcoholism and other socio-economic problems.^20^

Gender-based violence (GBV) in South Africa is amongst the highest in the world.^21^ Contributing and causative factors stem from the influences of culture, tradition and religion but the socioeconomic impacts of poverty, alcohol abuse, gun-violence and poor access to legal services are also major role players.^22^ In addition to the physical and behavioural consequences of GBV, post-traumatic stress disorder (PTSD), major depression and generalised anxiety disorders are documented in victims.^22^ Domestic abuse or rape was reported in five of our subjects, and psychiatric diagnoses of PTSD, depression and anxiety were also documented. This may aid in understanding why FNDs are more prevalent in the female population in South Africa.

Older studies in Africa have documented the influence of cultural beliefs on mental health presentations.^23^ Such beliefs are commonly observed in the local population, and many patients may seek the help of traditional healers or ‘sangoma’.^24^ We identified only two subjects whose family members believed that symptoms were because of witchcraft; however, it is possible that many were not directly interrogated on their beliefs or accessing traditional medicine.

A high rate of psychiatric comorbidities including depression, anxiety, post-traumatic stress and personality disorders have been documented locally and internationally in patients presenting with PNES.^19,25,26,27^ This corresponds to our finding that a range of psychiatric conditions are prevalent amongst patients with FND, with depression being most common.

Our study also examined the overlap with organic disease, with hypertension being most commonly followed by HIV, asthma and diabetes mellitus; however, no significant correlations were found. Given the high prevalence of HIV in Southern Africa, this may reflect an under-recognised aspect of the neuro-psychiatric consequences of the HIV epidemic in sub-Saharan Africa. It is possible that the stress of being diagnosed with HIV or HIV-related neurocognitive impairment may contribute to the development of FNDs, but an incidental correlation between the two is also likely because of the high burden of HIV in South Africa.

In the 2018 Tanzanian case series and 1-year long retrospective review in the United States, the most common FND phenotypes reported were PNES and gait impairment, respectively.^12,16^ Our study did not support the predominance of the PNES phenotype as sensory disturbance was the most common neurological complaint, followed by weakness, seizures and gait dysfunction.

We identified a predominance of paraparesis when patients were weak, hemisensory loss when patients had sensory symptoms and tremor when patients had a movement disorder. Tremor, being one of the most common forms of functional movement disorder, has been documented previously.^28^

Previous reviews have reported a predilection for the left in up to 60% of patients presenting with functional weakness and sensory symptoms.^5,29^ Over half of the hemiparetic subjects in our study had a left-sided weakness.

Multiple somatic symptoms such as headache, pain and fatigue are found in patients with FND. Irritable bowel syndrome (IBS), chronic fatigue syndrome, fibromyalgia and chronic pain syndromes are also documented.^30^ Many of these symptoms were found in our patient sample, with headache being the most commonly documented. Specific diagnoses of IBS, chronic fatigue syndrome and fibromyalgia were not documented; however, pain was a frequently reported symptom suggesting that such functional pain syndromes may have been present.

The impact of family members’ illnesses on phenotypic presentations of FND, or ‘modelling’ as described by Stone et al.,^5^ was corroborated by our findings and in particular those who presented with PNES.

To distinguish FNDs from organic pathology, ‘positive functional signs’ may be found on examination. Some of these signs include inconsistency, intermittency, variability, Hoover’s sign, distractibility and the ‘hemisensory syndrome’. The commonest signs found in FNDs have not been documented locally. Inconsistency, the most frequently documented sign in our study, refers to discrepancies in findings but does not differentiate whether this is conscious or unconscious.^5^ A patient may be able to walk but, when examined supine, is unable to lift his or her leg off the bed. Intermittency was documented in 15.8% of our subjects and describes the power that ‘comes and goes’ but is normal with encouragement.^5^ Variability, whether of frequency, amplitude or distribution was the third most common clinical finding in our study, and not only in relation to movement disorders as is often observed.^5^ Hoover’s sign, which demonstrates the discrepancy between voluntary and involuntary hip extension, is the only sign found in controlled studies to have a high specificity (100%; sensitivity 63%).^31,32^ It was only documented in 5.7% of our subjects. Distractibility, or improvement in abnormal movements when performing tests of mental concentration,^5^ was also documented infrequently. The ‘hemisensory syndrome’, recognised for over a century, refers to the splitting of sensation down the midline.^33^ However, in subjects with FND, vibration sense is also discrepant on either side of the sternum or frontal bone despite each vibrating as single units, a distinguishing feature.^5^ This was observed in six subjects. Not all ‘positive functional signs’ that have been documented in previous studies were assessed in our patients.^5^ Examining the sensitivity and specificity of particular signs prospectively are a consideration for future studies in South Africa.

A number of considerations arise when investigating functional disorders. Investigations are performed to exclude organic pathology but some studies propose neuroimaging to support the diagnosis of FND. This includes the emerging theory of a ‘software problem’, which describes abnormal neurobiological mechanisms (‘software’) despite an intact macroscopic brain structure (‘hardware’).^34^ A recent systematic review highlighted that individuals with FND and somatic symptom disorders (SSD) might exhibit sensorimotor, prefrontal, striatal-thalamic, paralimbic and limbic structural alterations and therefore may have both a ‘software’ and ‘hardware’ problem.^34^

In resource-limited settings, however, the exclusion of organic pathology overrides proving a pathophysiological basis of FND. For example, the 2018 study in northern Tanzania had access to a single CT scanner and EEG machine with the closest MRI facility being approximately 80 km away.^12^ As our study reviewed patients attending a tertiary-level hospital with most investigations being readily available, this may not be representative of most hospitals in South Africa where access to specialist care, neuroimaging and neurophysiological studies is very limited, if available at all. This emphasises the need for clinical education on FNDs to ensure that resources are utilised cost-effectively, without compromising the quality of patient care.

In an audit conducted in the United Kingdom by Parry et al.,^17^ the number of investigations carried out and resource utilisation contributed to high costs incurred as a result of FNDs. The authors found that the EEG was the commonest study requested. This is in contrast to the present study, in which MRI was the most frequently performed investigation.

Cerebrospinal fluid analysis was the least frequently performed investigation analysed in our study and did not aid in diagnosis. Besides the attendant cost, LPs are invasive tests with procedural complications, and in patients with underlying psychiatric illnesses they may precipitate further somatic symptoms. This is another reason, besides cost, why limiting unnecessary investigations in patients with FNDs is crucial.

Neurophysiological studies other than the EEG, such as motor-evoked potentials (MEPs) and Bereitschaft potentials, may assist in further defining pathophysiology in FNDs.^35^ These modalities were not analysed in our study as apart from being rarely performed (*n* = 4/158), their use in the context of FND in South Africa has not been studied.

Management of FNDs remains challenging, as demonstrated by the high proportion of subjects in our study who showed little or no improvement on follow-up. Whilst data on optimal management strategies are limited, there is evidence that a multidisciplinary team (MDT) approach is effective. This was shown in studies of inpatients with severe and chronic FNDs.^36,37^

The benefit of rehabilitative physiotherapy, demonstrated in a randomised control study,^38^ has been underutilised locally, with only 13.3% of subjects in our study having received physiotherapy. This may have been because of the fact that sensory disturbances were the most common functional symptom documented in our study and clinicians may have been unaware of the benefit of physiotherapy even in these patients. Another consideration is that clinicians themselves did not believe that their patients’ symptoms were ‘real’ and therefore did not warrant physiotherapy.

The majority of subjects were referred for or received psychotherapy (72%) and a smaller proportion were commenced on antidepressants (17.4%). This represents a higher proportion of patients with FND referred to psychiatric services than in a United Kingdom audit.^17^

Psychotherapy, in particular, cognitive behavioural therapy (CBT) has shown benefit in the management of PNES compared with standard medical care.^39^

Access to psychological support services may vary between the private and public sectors in South Africa. Patients attending an epilepsy-monitoring unit at a private hospital facility were noted to have access to an FDN integrating the work of psychologists, psychiatrists and neurologists,^18^ whilst a local public sector study on PNES reported that only 30% of patients received psychotherapy.^19^ The establishment of similar networks in the public sector would be a major advance in the management of FND and encourage a multidisciplinary approach.

Emerging data on the links between emotional states and FNDs have implicated autonomic dysregulation, abnormal limbic motor interactions and abnormal bodily awareness.^1^ An increasing awareness of ‘mind–body’ connections has prompted renewed interest in this area, and relaxation techniques such as ‘mindfulness’ and meditation may hold promise in the future management of functional disorders.

Therapeutic sedation has been used with some benefit in a case series although it remains a contentious form of treatment raising ethical concerns.^40^

Newer treatment modalities such as transcutaneous electrical nerve stimulation (TENS) and transmagnetic stimulation (TMS) have not been described in the present study or other local studies, and whether such data from the developed world can be extrapolated to the South African context remains unknown.^41^

The prognosis of patients with FNDs on long-term follow-up appears to be guarded. We found that of the subjects who were followed up in our study, the majority had no improvement in their symptoms and 17% reported deterioration at follow-up. This is in keeping with international data on the outcomes of functional motor disorders and PNES; however, these studies were limited by a lack of validated outcome measures.^42^

A recent review by Pick et al.^43^ addressed the inconsistency of outcome measures and concluded that few are well validated such as the Functional Movement Disorder Rating Scale (S-FMDRS)^44^ and the Conversion Disorder Scale (CDS).^45^ Future studies that apply validated outcome measures will be of benefit to patients and clinicians.

Interest in the complex field of FNDs in South Africa is growing, and as more data on this subject becomes available, current practice will continue to evolve. Lack of training in the identification and management of patients with FNDs is noted to be a barrier to patient care; therefore, raising awareness amongst clinicians is an area of particular concern.^19^ Our study presents the spectrum of FNDs seen at an academic centre in South Africa, and we anticipate that future studies will continue to expand our knowledge and proficiency in this area. As recent studies have suggested, there is a need to eradicate the stigma attached to the diagnosis, to patients and their clinicians and reform the approach taken in managing this condition.^7^

## Strengths

Although there have been reports of FND in Africa since the 1950s,^13^ this study is the first in South Africa to assess the full clinical spectrum of FND, rather than a subset of patients. This is also the largest study conducted on FND in Africa to date. The electronic database available at the hospital provided a reliable, secure data source. The study was conducted in the public healthcare setting, which is accessed by the majority of patients in South Africa and is, therefore, likely to be representative of the general population.

## Limitations

This was a retrospective study that did not employ specific outcomes measures, and some patient data were missing. Our study was also limited to a specific geographical area and to a single tertiary referral centre although patients were referred to this facility from throughout the province of KwaZulu-Natal.

## Conclusion

Whilst the spectrum of FND is broad and heterogeneous, our study found the most common phenotype to be sensory impairment. A significant proportion of those with PNES had a family history of epilepsy, supporting the theory of ‘modelling’.^5^ The majority of subjects were female, unmarried, unemployed and of black African ethnicity and the impact of cultural and socioeconomic factors requires further study. Comorbid psychiatric disease and clinical signs such as inconsistency may be useful in establishing the diagnosis of FND. Neuroimaging and neurophysiological studies play a role in the exclusion of organic pathology but should be individualised in a resource-limited setting. The prognosis of patients in this context was poor, but evidence suggests a multidisciplinary approach may offer better outcomes. Further research in Africa is required to expand current knowledge and expertise in this area, reduce stigma and improve both clinician awareness and patient care.

### Abnormal findings on neuroimaging

Abnormal MRI findings included cerebral atrophy, cerebellar atrophy, small vessel disease, sinusitis, periventricular non-specific lesion, chronic lacunas, degenerative spine disease, disc protrusions, old infarcts and an incidental pineal cyst. Only one patient had mesial temporal sclerosis reported on MRI; however, this patient was known with epilepsy and the reason for presenting was a hemiparesis rather than seizures.

Computed tomography brain findings included hemiatrophy, cerebellar atrophy, lacunar infarcts, incidental granulomas, calcification, arachnoid cyst, prominent pituitary gland and bilateral basal ganglia hyperintensities.
